# The T Cell Receptor Immune Repertoire Protects the Liver From Reconsitution

**DOI:** 10.3389/fimmu.2020.584979

**Published:** 2020-12-18

**Authors:** Qing Liang, Yudi Hu, Meina Zhang, Chunjie Lin, Wei Zhang, Ying Li, Ping Zhu, Pengxin Xue, Yujie Chen, Qiyuan Li, Kejia Wang

**Affiliations:** ^1^National Institute for Data Science in Health and Medicine, School of Medicine, Xiamen University, Xiamen, China; ^2^State Key Laboratory of Cellular Stress Biology, Innovation Center for Cell Signaling Network, School of Life Sciences, Xiamen University, Xiamen, China; ^3^Department of Pathology, The 971 Hospital of People’s Liberation Army Navy, Qingdao, China; ^4^Department of Pathology, Qingdao Municipal Hospital, Qingdao, China; ^5^Department of Gynaecology and Obstetrics, The 971 Hospital of People’s Liberation Army Navy, Qingdao, China

**Keywords:** T cell receptor, immune repertoire, liver fibrosis, mass cytometry, intrahepatic immune microcircumstance

## Abstract

Aberrant immune cell infiltrates and microcircumstances represent characteristic features of liver fibrosis. In this study, we profiled the transcriptomes of intrahepatic CD45^+^ immune cells, from mice, using single-cell RNA sequencing (scRNA-seq) technology to understand the landscape of intrahepatic immune cells during the pathogenesis of fibrosis. Analysis of approximately 10,000 single-cell transcriptomes revealed an increase in dendritic cells (DCs), macrophages, and neutrophils and a decrease in T and natural killer T (NKT) cells. In addition, we report changes in the transcriptomes of diverse immune cell types, implying a deteriorating intrahepatic immune microcircumstance. Furthermore, we uncovered a novel fibrosis-associated CD8 T (*Ccl5*^+^, *Ccl4*^+^) and CD4 T (*mt-Co1*^+^) cell subpopulation, which infiltrates fibrotic liver and is characterized by abnormal activation or inactivation as well as a TCR decline. The results from scRNA-seq and bulk immune repertoire sequencing (IR-seq) revealed an obvious decline in T cell receptor (TCR) clonotypes combined with shrinking VJ and VDJ segment usage, as well as lower complementarity-determining region 3 (CDR3) amino acid (AA) diversity from fibrotic liver. Interestingly, a deficiency of TCR IR (Tcrb^KO^ mice) led to a deterioration of liver fibrosis, coupled with activation of hepatic stellate cells (HSCs) induced by the upregulation of macrophage and γδ T cell distribution in fibrotic Tcrb^KO^ livers. Our findings reveal the landscape and dynamics of single immune cells in liver fibrosis, and clarify the protective role of TCR IR in response to chronic liver injury.

## Introduction

Liver fibrosis, a common final pathway for most chronic liver diseases, poses a threat to public health worldwide, accounting for more than 1 million deaths each year (1, 2). The condition mainly results from chronic liver inflammation, often induced by virus-associated hepatitis, drug/alcohol abuse, or autoimmune liver disease, and features excess accumulation of extracellular matrix subsequent to chronic liver injury (3–5). Accumulating evidence has revealed the critical role of activated hepatic stellate cells (HSCs) in collagen and extracellular matrix (ECM) production (3, 6, 7). Upon liver injury, HSCs, the major source of collagen in the liver, transdifferentiate from quiescent lipocytes into myofibroblast-like cells to drive fibrogenesis, which is characterized by enhanced chemotaxis, survival and collagen production (4, 8, 9). Despite their importance in liver wound healing, it is hypothesized that activated HSCs may encourage pathogenesis during liver fibrosis.

The liver is a central immunologic organ, known to induce immune tolerance and immunity (10). Abundant immune cells in the liver function to maintain intrahepatic immune homeostasis. Recent studies have emphasized the crucial role played by different intrahepatic immunocyte subsets, especially macrophages, in the progression of liver inflammation and fibrosis (11–15). Activation of HSCs depends on the intrahepatic immune microcircumstance, including immune cells (T, B, natural killer (NK) and macrophages cells) and signaling in an autocrine and/or paracrine fashion (16–19). Recent studies have described the role of T cells, including γδ T cells, Th17 cells and natural killer T (NKT) cells, in modulating inflammation and fibrosis in response to liver injury (17, 20, 21). However, their contributions to HSC activation as well as the molecular mechanisms of T cells in fibrotic pathogenesis remain largely unknown.

In our previous work, we demonstrated that TCR IR reconstitution acts more like a “commander” than an “executor” in the intrahepatic immune microenvironment (22), indicating that it contributes not only to TCR-mediated recognition and pathogen clearance, but also to tissue immunosurveillance and immunoregulation. However, the TCR-mediated precise connection between the TCR clonotype and the transcriptomic state of intrahepatic T cells remains largely unknown. In the current study, we applied scRNA-seq to characterize CD45^+^ immune cells from different phases of fibrotic liver. Combining transcriptomic profiles with TCR usage revealed the emergence of distinct activated T cell subsets as well as associations between T cell state and TCR transformation in liver fibrogenesis. We limited the present analysis to determining a functional link between TCR IR and liver fibrosis. Overall, our results demonstrate the regulatory role of TCR IR in the intrahepatic immune microcircumstance and provide a novel viewpoint of the role of T cells in liver fibrogenesis.

## Materials and Methods

### Animals and Fibrotic Models

WT C57BL/6 and TCRβ^KO^ mice were housed in pathogen-free animal rooms at the Animal Care Centre of Xiamen University (Xiamen, China). All animal experiments were approved and conducted under the Guidelines of the Xiamen University Committee on Animal Care and Use. For liver fibrosis, 6- to 8-week-old male mice were injected intraperitoneally with carbon tetrachloride (CCl_4_, dissolved in olive oil with a volume ratio: 1:1) (Sigma-Aldrich, St. Louis, MO, USA) at a dose of 1 μl/g body weight thrice per week for 4 or 8 weeks. Samples were collected 1 day after the last injection. Bile duct ligation (BDL) and sham laparotomy were conducted as described previously (23). The 2-week time point was chosen to study liver fibrosis.

### Preparation of Single-Cell Suspensions and scRNA-Seq

The isolation of mouse intrahepatic immunocytes was performed as previously described (22). Based on FACS analysis, single CD45^+^ cells (1 × 10^6^ cells, 90%–95% viability, four mice per sample) from untreated, 4- and 8-week CCl_4_-treated livers were sorted into 1.5 ml tubes with 50 ml sorting buffer. For droplet-based scRNA-seq, the cells were loaded onto a 10X Genomics Chromium chip (10X Genomics, Pleasanton, USA) as per the factory recommendations. Reverse transcription and library preparation were performed using the 10X Genomics Single Cell v2 kit following the manufacturer’s protocol. For single-cell VDJ sequencing, the cDNA encoding the TCR library was amplified with two-round PCR using the DNA primers provided in the kit, followed by selection with SPRI beads (Bulldog Bio Inc. New Hampshire, USA). The libraries were sequenced on an Illumina HiSeq 4000.

### Statistical Analysis

All statistical data were analyzed using the GraphPad Prism 8.0 Package (GraphPad Software, La Jolla, CA). Data are presented as the mean ± standard deviation (SD). Student’s t-test was used to compare values between two groups. Differences among multiple groups were determined using one-way analysis of variance (ANOVA) followed by Sidak’s multiple comparisons test for mean separation. Statistical significance was accepted at *P*<0.05.

For further details regarding the materials and methods used, please refer to the **Supplementary Materials**.

## Results

### scRNA-Seq Reveals an Aberrant Immune Microcircumstance in Fibrotic Liver

**Figure 1:**
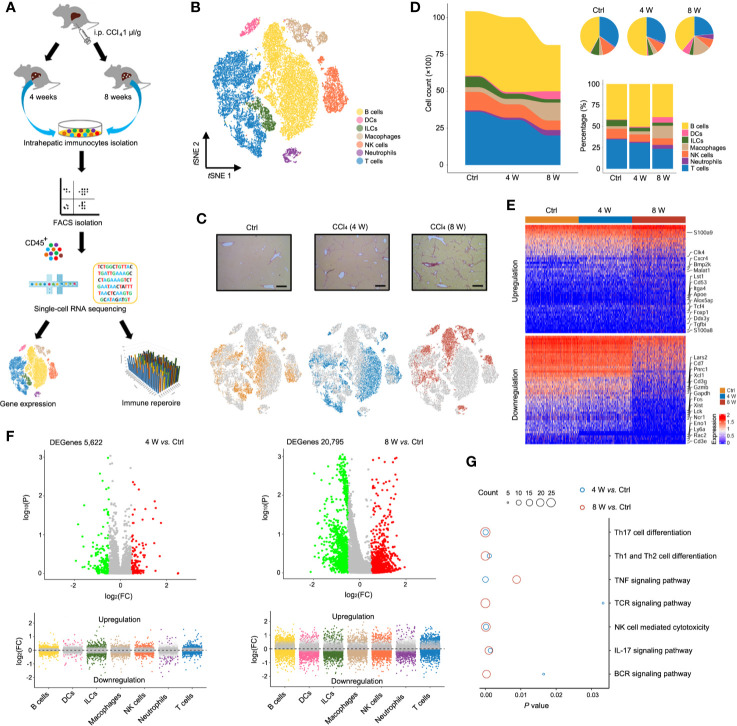
Single-cell atlas of intrahepatic immune cells. **(A)** Flow chart of scRNA-seq and IR-seq. **(B)** scRNA-seq of intrahepatic immune cells from untreated and 4- and 8-week CCl_4_-treated livers. *t*SNE clustering of 28,608 single-cell transcriptomes (Ctrl: 10,436 cells, 4-week: 10,028 cells and 8-week: 8,144 cells, n = 4) colored according to cell-type clusters. **(C)** Representative Sirius red staining of untreated, 4- and 8-weeks CCl_4_-treated livers (up). *t*SNE clustering as in b, but colored according to groups (down). **(D)** The number and percentage of each cell type. **(E)** Heatmap showing Z-scored mean expression of differentially expressed genes (DEGenes top 50). **(F)** A volcano plot showing DEGenes among the groups (up). Liver fibrosis-dependent gene expression in each cell type (down). Green dots indicate significantly decreased DEGenes, while red dots indicate significantly increased DEGenes. **(G)** Pathway enrichment for the DEGenes among the groups. Circle size indicates enrichment gene number (complete pathway enrichment available in **Table S3**).

To characterize the state of immune cells during liver fibrosis, we performed scRNA-seq on CD45^+^ cells isolated from untreated (Ctrl) and CCl_4_-induced 4 (4 W) and 8-week (8 W) livers. For TCR IR analysis, both scRNA-seq and IR-seq methods were applied simultaneously to evaluate TCR transformation during liver fibrosis (**Figure 1A**). We obtained expression profiles for 10,436, 10028, and 8,144 pure CD45^+^ cells from the Ctrl, 4 W and 8 W groups, respectively (**Figure S1A**). Analysis of single-cell transcriptomes using *t* distributed stochastic neighbor embedding (*t*SNE) projection revealed 7 distinct immunocyte subsets in the liver (**Figure 1B**). Characterization of their markers indicated that they were B cells, dendritic cells (DCs), innate lymphoid cells (ILCs), macrophages, NK cells, neutrophils, and T cells (**Figure S1B**, **Table S1**). Notably, we observed a shift in the gross number of cells from normal to fibrotic livers (**Figure 1C**). As expected, fibrogenesis was accompanied by an aberrant cell distribution (**Figure 1D**) as well as shifts in the transcriptomic states of various cell types (**Figures 1E, F** and **Figures S1C, D**, **Table S2**). Notably, the total cell number and percentage of macrophages expanded to orchestrate liver fibrosis progression, as has previously been reported (14). Furthermore, the results from KEGG analysis indicated enrichment of differential genes in immune signaling pathways, including TNF-α, IL-17, NF-κb, TCR, and BCR signaling (**Figure 1G**, **Table S3**). In summary, analysis of scRNA-seq data resulted in the identification of multiple immune cell populations with aberrant distribution patterns and transcriptomic states during liver fibrosis.

### Fibrogenesis Diminishes the Intrahepatic T Cell Population

**Figure 2:**
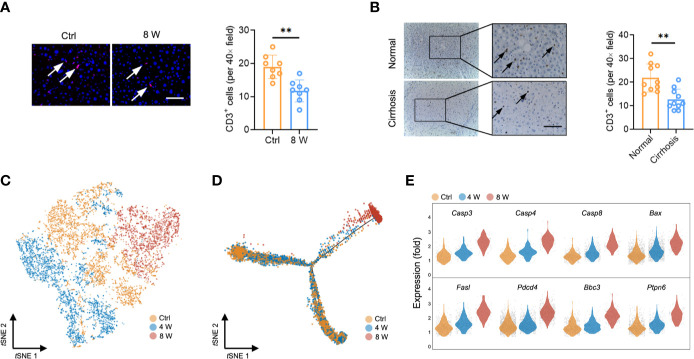
T cell reduction during liver fibrosis. **(A)** Representative images of immunofluorescence results of CD3 in untreated and 8-week CCl_4_-treated livers (n = 8, **P < 0.01). Scale bar, 100 μm. **(B)** Representative images of immunohistochemistry results of CD3 in human normal and cirrhotic livers (n = 10, **P < 0.01). Scale bar, 100 μm. **(C)** *t*SNE clustering of 8,573 single-cell transcriptomes (T cells) colored by groups. **(D)** Trajectory analysis for T cell scRNA-Seq data in a two-dimensional state-space inferred by Monocle 2. **(E)** Normalized expression values of 8 apoptotic genes (*Casp3, Casp4, Casp8, Bax, Fasl, Pdcd4, Ptpn6*, and *Bbc3*) in T cells.

Interestingly, we found a reduction in the T cell population (earlier observed in **Figure 1D**) in fibrotic liver (both in CCl_4_-induced liver and BDL-induced liver) based on the results from immunofluorescence staining (**Figure 2A** and **Figure S2A**). Livers from cirrhotic patients also exhibited a reduction in CD3^+^ cells, indicating that the reduction of the T cell population in fibrotic liver occurs in both mice and humans (**Figure 2B**). To characterize the T cells during fibrosis, we used *t*SNE to visualize the T profiles from the single-cell transcriptomic data. Notably, we found that T cells from Ctrl (3606 cells), 4 W (3052 cells), and 8 W (1915 cells) generally partitioned into distinct areas, suggesting a difference between T cells in fibrotic liver and those in the Ctrl (**Figure 2C**). To clarify the T cell transition, we used the Monocle method to construct potential developmental trajectories of the four T cell clusters based on the expression data (**Figure S2B**). The developmental trajectories revealed distinct functional states in the late fibrosis phase, with T cells from 8 W positioned at the opposite end of those from Ctrl, and T cells from 4 W located in between, indicating intermediate functional states (**Figure 2D**). In rodent models, HSC-induced T cell hyporesponsiveness is associated with enhanced apoptosis (24, 25). Analysis of scRNA-seq data from the T cells revealed a significant upregulation of apoptosis-related genes in fibrotic liver, including *Casp3, Casp4, Casp8, Bax, Fasl, Pdcd4, Ptpn6*, and *Bbc3* (**Figure 2E**, **Figures S2C, D**). Overall, these findings indicated that intrahepatic T cells, widely experiencing apoptosis, may partly account for the T cell population shrinkage.

### Intrahepatic T Cell Clusters Exhibit Diverse Patterns

**Figure 3:**
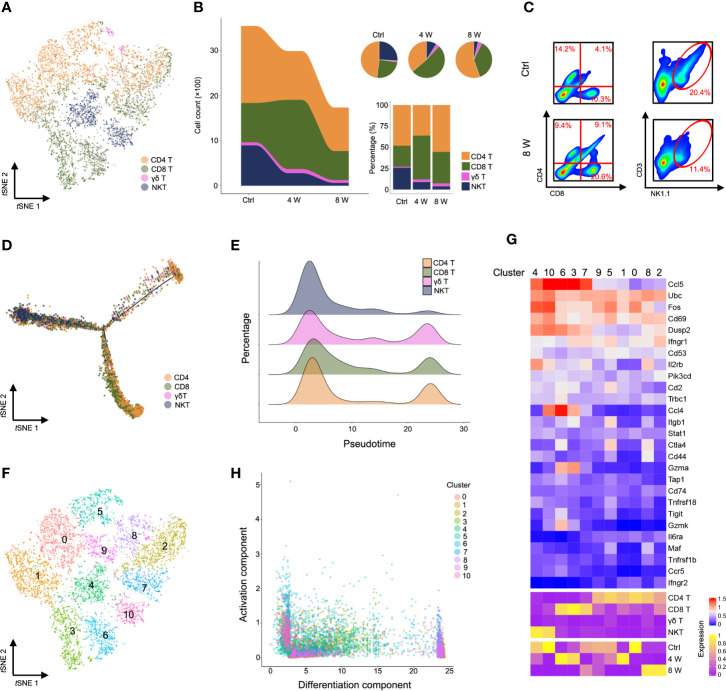
Characterization of T cells during liver fibrosis. **(A)** *t*SNE clustering as in c, but colored by T cell type (CD4 T, CD8 T, γδ T, and NKT). **(B)** The number and percentage of T cell types. **(C)** Flow cytometry for CD4 T cells (CD4^+^), CD8 T cells (CD8^+^) and NKT cells (NK1.1^+^, CD3e^+^) in untreated, 8-week CCl_4_-treated livers. **(D)** Trajectory analysis of CD4 T, CD8 T, γδ T and NKT cells. **(E)** Percentage of four T cell clusters inferred by Monocle’s pseudotime ordering as in d. **(F)** T cells identified using unsupervised clustering based on *t*SNE. **(G)** Heatmap showing the expression of immune-activated genes with Z scores standardized across each cluster, cell type and group (complete data available in **Table S7**). **(H)** Visualization of all T cell clusters using the activation component and differentiation component. Each dot represents a cell colored by cluster.

Characterization of markers identified CD4 T, CD8 T, γδ T, and NKT cells (**Figure 3A**, **Figure S3A**, **Table S4**). Strikingly, single-cell transcriptome data and fluorescence activated cell sorting (FACS) revealed a gradually diminishing NKT cell population, while CD8 T cells were expanded in fibrotic liver (**Figures 3B, C** and **Figure S3B**). Similarly, NKT cells were highly enriched in the early pseudotime period (**Figures 3D, E****)**, demonstrating the protective roles of NKT cells in liver injury models (26, 27). Furthermore, we observed an expansion of CD8 T cells in the middle of the pseudotime (**Figures 3D, E****)**, confirming that these cells invade into fibrotic liver to promote fibrosis (28). To investigate the role of T cell subpopulation, αβ T cells were classified as native T cells, helper T cells, cytotoxic T cells (CTL), regulatory T cells (Treg), exhausted T cells, memory T cells, proliferative T cells according to cell marker (**Figures S4A, B**). Notably, a gradually expanded Treg cell population was found in liver fibrosis (**Figures S4C, D**). The variation of gene expression in Treg were showed in **Figures S4E, F**, **Table S5**. Our results were consistent with previous studies, suggesting that liver fibrogenesis expands liver Treg, which reduce the function of bulk intrahepatic T cells yet limit liver injury (29, 30). To further understand the T cell heterogeneity during liver fibrosis, we performed unsupervised clustering, based on *t*SNE, and identified 11 cell clusters (**Figure 3F**, **Figure S3C**, **Table S6**). Notably, we identified highly activated clusters from the Ctrl and 4 W groups. These included clusters 4 and 10 (higher expression of *Ccl5*^+^, *Fos*^+^, *Dusp2*^+^) and clusters 6, 3, and 7 (higher expression of *Ccl5*^+^, *Ccl4*^+^), which expressed several markers of NKT and CD8 T cells. Clusters 8 and 2 exhibited higher expression of *mt-Co1* with lower activation of infiltrates in late-phase liver fibrosis (**Figures 3G, H**, **Figure S3D**, **Table S7**). Taken together, these results indicated that activation of NKT cells is essential for hepatic homeostasis. Liver injury gives rise to CD8 T cell infiltration and activation; therefore, inactivating CD4 T cells leads to liver fibrosis.

### TCR Clonotypes Show a Lower Diversity in Liver Fibrosis

**Figure 4:**
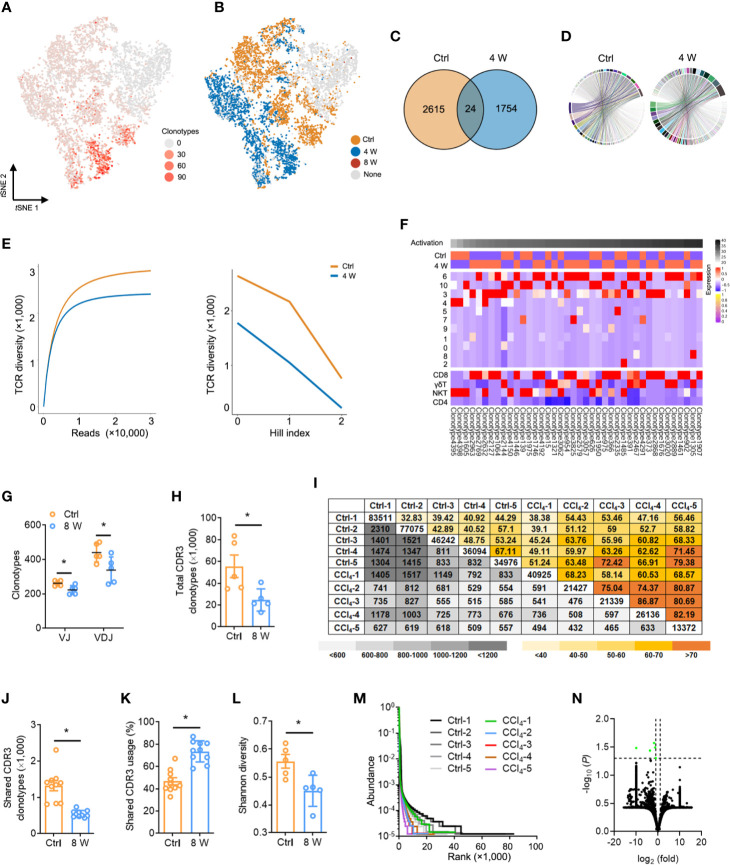
Fibrogenesis shapes shrunk TCR repertoires. **(A)** *t*SNE plot colored with single-cells with a high frequency of TCR clonotypes shown in red, and those with a low frequency are shown in grey (complete data available in **Table S8**). **(B)** *t*SNE plot of TCR clonotypes colored by groups. **(C)** Venn diagrams showing the number of TCR clonotypes in scRNA-seq data from untreated and 4-week CCl_4_-treated livers. **(D)** The composition of the VJ combination. The color indicates distinct V and J segments. **(E)** Dilution curve (left) and Hill index (right) showing TCR diversity. **(F)** A heatmap showing the expression pattern of the top 40 TCR clonotypes with Z scores standardized across each cluster, cell type, and group (complete data available in **Table S8**). **(G)** TCRβ IR-seq of intrahepatic immune cells from untreated and 8-week CCl_4_-treated livers. Clonotypes of TCRβ VJ and VDJ combination. **(H)** A total of TCRβ CDR3 AA clonotypes in each group (complete data in **Table S11**). **(I)** Quantification (grey plots) and frequency (colored plots) of intrahepatic TCRβ CDR3 AA clonotypes. Uncolored plots indicate the total clonotypes per sample. **(J)** The shared TCRβ CDR3 AA clonotypes in each group. **(K)** The percentage of shared TCRβ CDR3 AA clonotypes in each group. **(L)** Shannon diversity showing TCRβ CDR3 AA diversity. **(M)** Rank-abundance curve showing TCRβ CDR3 AA richness and evenness. **(N)** A volcano plot showing TCRβ CDR3 AA clonotypes. Green dots indicate significantly downregulated TCRβ CDR3 AA clonotypes. In each experimental group, five to 10 mice were used. *P < 0.05.

Studies have reported that a diverse repertoire of TCR leads to a continuous spectrum of T cell activation, obscuring the transitional states (31). To investigate whether TCR diversity variation contributed to T cell inactivation, we performed scRNA paired V(D)J sequencing of T cells in the same sample (**Table S8**). In general, we observed less TCRα, TCRβ, and TCRαβ during liver fibrosis (**Figure S5A**), while T cell clusters with high activation were significantly expressed in diverse TCR clonotypes, similar to that seen in **Figure 3G** (**Figure 4A**). Conversely, almost no expression of TCR was recorded in T cells from late-phase fibrotic liver (**Figure 4B**). Consequently, we analyzed the V(D)J sequencing data of cells from the Ctrl and 4 W groups and found a significant decrease in TCR clonotypes from fibrotic liver relative to those in the Ctrl group (**Figure 4C** and **Figure S5B**), although the compositions of the VJ and VDJ clonotypes showed no differences (**Figure 4D**, **Figure S5C**, **Table S9**). TCR diversity and their abundance in fibrogenesis were significantly constrained (**Figure 4E**). To gain deeper insights into the characteristics of the TCR clonotypes, we focused on the top 40 TCR clonotypes and found enrichment of high-frequency TCR clonotypes in T cell clusters (clusters 6, 10, 3, and 4) with activation, which belonged to CD8 T, γδ T, and NKT cells. On the other hand, CD4 T cells exhibited fewer high-frequency TCR clonotypes (**Figure 4F**). These findings supported the idea that high-frequency TCR clonotypes contribute to T cell activation during fibrogenesis.

We further assessed the distribution and diversity of TCR using bulk TCRβ IR-seq from untreated and 8 week CCl_4_-induced livers and identified a total of 25 V, 13 J, 290 VJ, and 551 VDJ segments from all samples (**Table S10**). Usage patterns for most V, J, VJ, and VDJ segments were similar between the two groups, whereas only 2 V, 12 VJ, and 7 VDJ segments revealed significant usage differences (**Figure S6**). Strikingly, multiple VJ and VDJ combinations were absent during fibrogenesis, revealing shrunken and centralized VJ as well as VDJ combination usage in liver fibrosis (**Figure 4G** and **Figure S6E**). Given the importance of CDR3 AA clonotypes in determining the diversity of TCRβ IR, we identified 379,994 distinct CDR3 AA clonotypes (**Table S11**). Liver fibrosis led to obvious reductions in both total and shared CDR3 AA clonotypes, along with an increase in the percentage of shared CDR3 usage (**Figures 4H–K**). Similarly, Shannon diversity also exhibited remarkably diminished CDR3 AA diversity in liver fibrosis (**Figure 4L**). In addition, shrunken CDR3 AA richness and evenness were verified by rank-abundance analysis (**Figure 4M**), with the usage frequency of most CDR3 AA segments similar between the two groups (**Figure 4N**).

To explore the reconstitution of the TCR repertoire in fibrosis, we evaluated the differences in V-CDR3-J usage (**Table S12**). Overall, the TCR IR showed highly private clonotypes in the Ctrl liver, while several high-frequency CDR3 AA clonotypes were evident in 8-week CCl_4_-treated liver (**Figure S7A**). In addition, the percentage of the top 20 clonotypes was significantly higher in fibrotic liver, suggesting that fibrogenesis induced the usage of several key high-frequency CDR3 AA clonotypes (**Figure S7B**). We also assessed the frequency of terminal CDR3 AA motifs and found that fibrogenesis induced a higher frequency of terminal CDR3 AA motif glutamine-tyrosine-phenylalanine (QYF) as well as a lower frequency of terminal CDR3 AA motif leucine-tyrosine-phenylalanine (LYF) (**Figures S7C, D** and **Table S13**). Taken together, these results demonstrated a reconstitution of TCR IR during liver fibrosis, which leads to TCR diversity extinction.

### The Absence of TCR IR Leads to Susceptibility to Liver Fibrosis

**Figure 5:**
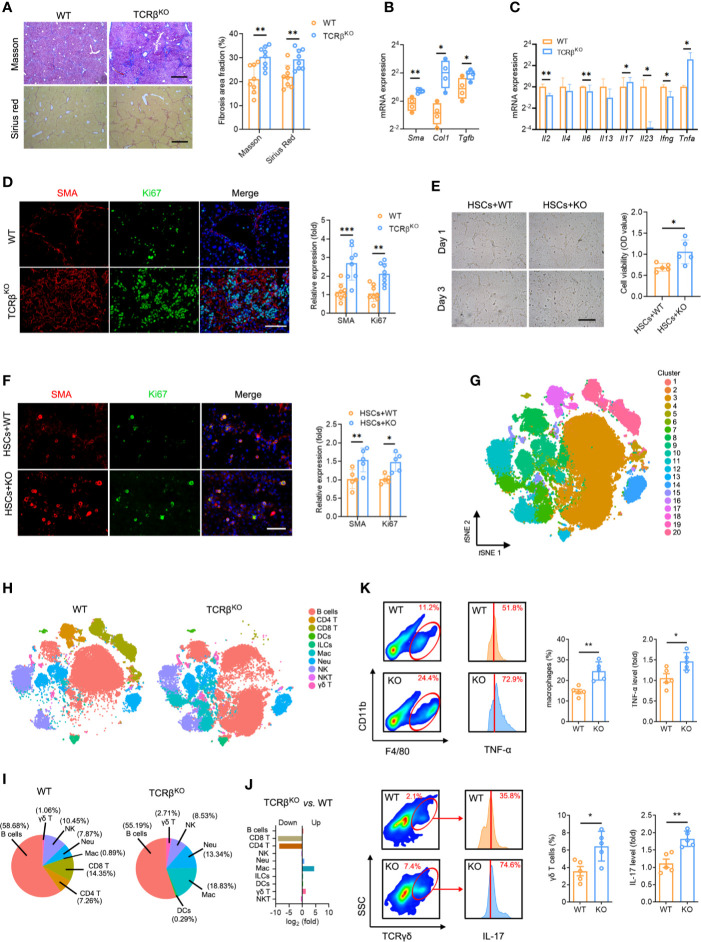
TCRβ deficiency aggravated CCl_4_-induced liver fibrosis. **(A)** WT and Tcrb^KO^ mice were treated 3 times weekly with CCl_4_ for 4 weeks. Representative Masson trichrome staining and Sirius red staining of liver tissues are shown. Scale bar, 200 μm. Quantification of the fibrosis area fraction in 10 cross-sections. **(B)** qPCR analysis of fibrosis-related mRNA levels of *Sma*, *Col1*, and *Tgfb* in the liver after CCl_4_ treatment. **(C)** qPCR analysis of inflammation-related mRNA levels of *Il2*, *Il4*, *Il6*, *Il13*, *Il7*, *Il23*, *Ifng*, and *Tnfa*. **(D)** Liver sections were stained for SMA (red) and Ki67 (green) to identify proliferating HSCs. Representative images are shown. Scale bar, 100 μm. Quantification of SMA and Ki67 expression in 10 cross-sections. **(E)** Representative images showing day 1 and day 3 cocultures of primary HSCs (isolated from WT mouse livers) with intrahepatic immune cell (isolated from WT or Tcrb^KO^ mouse livers after CCl_4_ treatment for 8 weeks). Scale bar, 50 μm. Cell viability was estimated using the CCK-8 assay after coculture for 3 days. **(F)** Immunofluorescence staining for SMA (red) and Ki67 (green) was performed to identify proliferating HSCs. Representative images are shown (left). Scale bar, 50 μm. Quantification of SMA and Ki67 expression. **(G)** Mass cytometry of intrahepatic immune cells from WT and Tcrb^KO^ mice (n = 4) after 4 weeks of CCl_4_ treatment (50,000 cells for each sample). *t*SNE plot showing all cells identified using unsupervised clustering. **(H)** *t*SNE clustering as in e, but colored by immune cell type. **(I)** The percentage of each immune cell in WT and Tcrb^KO^ after 4-week CCl_4_ treatment. **(J)** The ratio [log2(fold)] of distribution of each immune cell type. **(K)** Intrahepatic macrophages (F4/80^+^ and CD11b^+^, up) and γδ T cells (TCRγδ^+^, down) in the fibrotic liver detected by FACS (10,000 cells for each sample). Representative FACS plots are shown. Intrahepatic macrophages and γδ T cells harvested from fibrotic livers were gated and used to test for the expression of TNF-α (up) and IL-17 (down). Approximately four to eight mice were used in each experimental group. *P < 0.05; **P < 0.01; ***P < 0.001.

A key question is whether TCR-mediated T cell activation plays a function in liver fibrosis. Consequently, we subjected wild-type (WT) and Tcrb^KO^ mice to repetitive CCl_4_ treatments. We found that the livers of the control untreated adult Tcrb^KO^ mice were normal in appearance, size and weight, with no significant differences in architecture and inflammation compared to those from the control untreated WT mice (**Figures S8A–C**). The efficient deletion of TCRβ in the Tcrb^KO^ mice was confirmed by immunofluorescence analysis (**Figure S8A**). Masson staining and Sirius red assays revealed that TCRβ deficiency increased CCl_4_-induced liver fibrosis compared to WT mice (**Figure 5A**). Consistently, aggravated liver fibrosis development in Tcrb^KO^ mice was confirmed by elevated fibrogenic gene expression (*Sma*, *Col1*, *Tgfb*), along with abnormal intrahepatic immune status (including alleviated levels of *Il2*, *Il6*, *Il23*, *Ifng*, and enhanced levels of *Il17* and *Tnfa*) (**Figures 5B, C****)**. In addition, immunofluorescent staining revealed a high proliferation of HSCs in Tcrb^KO^ liver (**Figure 5D**). We, therefore, hypothesized that TCR-mediated T cell activation plays a critical role in intrahepatic homeostasis, which regulates the process of liver fibrosis.

### The Intrahepatic Immune Microenvironment, Not T Cells Alone, Modulates HSCs

To investigate the mechanisms by which TCR IR regulates hepatic inflammation and fibrosis, we cocultured primary HSCs (isolated from fibrotic liver) with or without T cells (isolated from normal liver) to verify whether T cells alone are responsible for the enhanced HSC proliferation. Interestingly, we found no differences in HSC proliferation and activation (**Figures S8D, E**), indicating that TCR IR is not the direct driver of HSC activation. In fact, other factors also contribute to this process. To explore the regulatory role of the intrahepatic immune microenvironment on HSCs, cocultures of WT mouse primary HSCs with intrahepatic immune cells, isolated from CCl_4_-treated WT mice or Tcrb^KO^ mice were performed for 3 days. CCK-8 assays and Ki67 immunostaining were performed to confirm HSC activation *in vitro*. Coculture with intrahepatic immune cells isolated from Tcrb^KO^ mice notably accelerated HSC growth and proliferation (**Figures 5E, F****)**. Together, these results demonstrated that HSC activation is modulated by the intrahepatic immune microenvironment rather than by T cells alone.

Due to the aberrant immune milieu in the fibrotic livers, we investigated whether TCRβ deficiency affects liver fibrosis by influencing the recruitment and activation of neighboring immunocyte subsets. We performed mass cytometry, targeting a global panel of 38 markers, and generated a detailed profile of the intrahepatic immune microcircumstance (**Figure S9A**). The results indicated the presence of 20 cell clusters using unsupervised clustering based on *t*SNE (**Figure 5G**, **Figure S9B**, **Table S14**). Furthermore, characterization of the markers revealed 10 distinct immunocyte subsets, including B cells, DCs, ILCs, macrophages, NK cells, neutrophils, CD4 T cells, CD8 T cells, γδ T cells and NKT cells in the liver (**Figure 5H**, **Figure S9C**, **Table S14**). Notably, Tcrb^KO^ livers displayed negligible T cell populations (both CD4 T and CD8 T cells) as well as expanded activated macrophages (cluster 7) and γδ T cells (cluster 15) in liver fibrosis (**Figures 5I, J****)**. FACS analysis also revealed significantly expanded macrophages and enhanced macrophages-labelled TNF-α levels. In addition, we observed an increased percentage of γδ T cells with elevated IL-17 levels in the fibrotic Tcrb^KO^ livers compared to WT mice (**Figure 5K**). Collectively, these results suggest that TCR IR deficiency selectively elevates TNF-α-producing macrophages and IL-17-producing γδ T cells, which leads to a deterioration of the immune milieu in the fibrotic liver.

## Discussion

The liver consists of parenchymal cells (hepatocytes and cholangiocytes), nonparenchymal cells (endothelial cells, biliary cells, and HSCs), and manifold immunocytes from the innate and adaptive immune system (32). Chronic liver injury typically provokes an aberrant intrahepatic immune microenvironment that is tightly modulated by the interplay of multiple pathways, molecules, and systems. It is dominantly dependent on interactions between damaged parenchymal cells, activated HSCs, and resident and infiltrating immune cells (33–36). Herein, we generated transcriptome data based on scRNA-seq. The data covered 28,608 individual CD45^+^ cells from the liver in different fibrogenesis stages, providing a rich resource for understanding the multidimensional characterization of immune cells during liver fibrosis. Consequently, using transcriptomic data, we identified a deteriorating intrahepatic immune microenvironment, along with aberrant cell distributions. However, macrophage/myeloid cell subsets will likely not be sufficiently discriminated due to the approach of CD45^+^ cell sorting.

Liver fibrosis is known to occur mainly through the hepatocyte-macrophage-HSC network (15, 37). However, there is also evidence for liver fibrosis occurring *via* direct interactions between immune cells and HSCs (7). The role of macrophages in liver cirrhosis has been studied in humans using scRNA-seq data. A study reported a decreased T-cell population (both CD4 T and CD8 T cells) in cirrhotic livers (14). In the present study, liver fibrogenesis resulted in a decrease in T cells *via* the upregulation of apoptosis-related genes in T cells, an observation that was consistent with previous findings (24, 38). We identified four distinct T cell subsets (CD4 T, CD8 T, γδ T, and NKT cells) and their states to further explore the transition of T cell subsets in fibrogenesis. Highly activated NKT cells were enriched in the normal liver, but faded away during liver fibrosis, suggesting that NKT might be a potential suppressor of liver fibrosis. A previous study suggested that CD8 T cells (*Ccl5*^+^, *Ccl4*^+^) might promote liver fibrosis *via* extrahepatic recruitment in response to liver injury (28). Additionally, the accumulation of ‘exhausted’ CD4 T cells (*mt-Co1*^+^) has been found in a terminal stage of liver fibrosis, indicating that CD4 T cells could be a negative regulator in liver injury and fibrosis (39).

The features of T cells are mainly shaped by TCR-mediated activation and TCR-dependent environmental exposures (31). Similar to T cell transition, a diminished diversity of TCR clonotypes has been observed during liver fibrosis, and a high frequency of TCR clonotypes is associated with activated T cell clusters, which suggests that TCR diversity could account for the continuous spectrum of T cell activation. Additionally, this continuity might be conducive for maintaining homeostasis *via* crosstalk between T cells and other immune cells. Emerging evidence suggests that TCR IR is associated with various liver diseases, including viral hepatitis, liver regeneration, hepatocellular carcinoma, primary sclerosing cholangitis, and alcoholic liver disease (40–43). Therefore, the identification and tracking of TCR IR may provide a novel strategy for investigating the dynamics and distribution of all T cell clonotypes and it represents a ‘footprint’ of immune status. In this study, we showed an intact characteristic of V, D, J, VJ, and VDJ segment usage and the distribution of CDR3 AA clonotypes in liver fibrosis. These results were consistent with scRNA-seq VDJ data, indicating that liver fibrogenesis reconstitutes TCR IR, leading to a remarkably lower TCR IR diversity. A restriction of the T cell immune repertoire was also found in chronic liver diseases and was related to limited antigen-driven T cell stimulation (40). Our work detected the precise connection between the TCR clonotype and the transcriptomic state of T cells by scRNA-seq analysis.

These TCR usage patterns, together with the specific TCR signaling associated with environmental exposures, jointly define the discrete states of intrahepatic T cells. Diverse TCR specificities, strong drivers of downstream transcriptional signaling, may alter the levels of transcripts associated with responses to environmental stimuli (31). Our characterization of the transcriptome data, coupled with detailed TCR-based lineage information, revealed the tight association between the TCR clonotype and transcriptional phenotype in liver fibrosis. In agreement with our observation regarding the accumulation of a highly aberrant immune milieu in TCRβ-deficient liver, it has been postulated that TCR reconstitution acts more like a “commander” than like an “executor” in the intrahepatic immune microcircumstance (22). However, the exact mechanism of the T cell interaction with other immunocytes remains unclear. In the present study, the ablation of TCRβ resulted in a significant exacerbation of liver fibrosis, as manifested by an aberrant immune milieu and HSC activation. Additionally, an unusual distribution of macrophages and γδ T cells combined with altered TNF-α and IL-17 levels was found in fibrotic TCRβ-deficient livers. The expanded amount of TNF-α-producing macrophages and IL-17-producing γδ T cells, which have been shown to promote hepatic fibrosis progression (44–46), may act as a feedback mechanism responding to TCR deficiency, thereby assuring aggravated hepatic fibrogenesis. The mechanism may be complicated. Multiple variables can impact the activation of macrophages and γδ T cells. According to pathway enrichment for DEGenes of scRNA-seq data, cytokines (*Ccl5, Ccl4, Ccl3, Gzmk, Gzma, Cxcr4, Il-4ra, Il10ra*) may be candidates for controlling pathogenic macrophages or γδ T cells.

**Figure 6:**
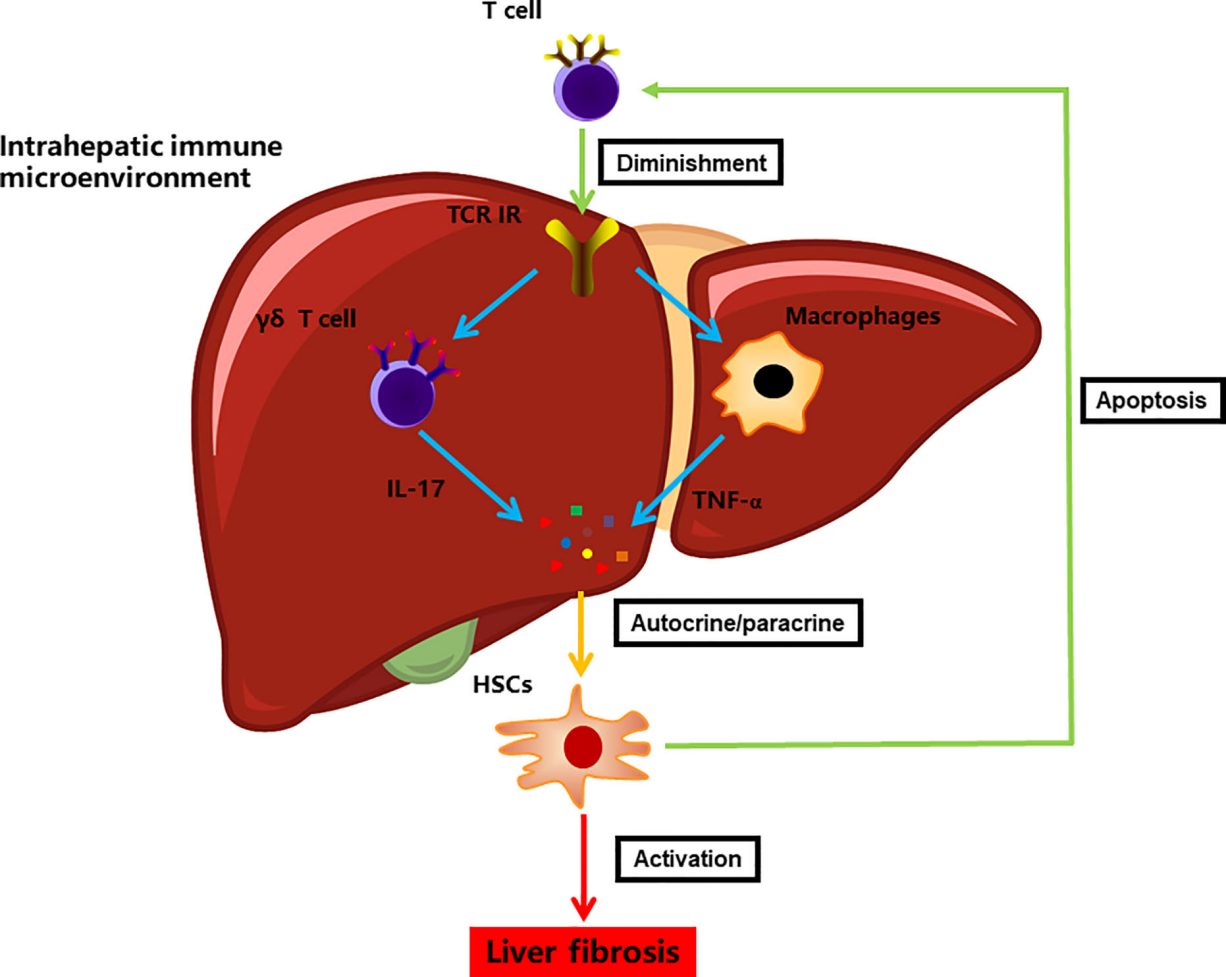
A model illustrating the immune microenvironment of liver fibrosis in the context of TCR IR.

The current study also elucidated the connection between TCR IR and HSCs in liver fibrosis, which revealed that TCR IR deficiency facilitated HSC activation. Notably, the coculture of HSCs with T cells did not affect HSC viability. This was consistent with our earlier speculation that the hepatic biological effect may be regulated by the intrahepatic immune microenvironment rather than solely *via* T cells (22). Although the nature of the crosstalk between HSCs and immune cells is still unclear, HSCs do receive many signals from individual immunocytes and, in turn, produce many fibrotic mediators that elaborate signals influencing the progression of liver fibrosis. Additionally, HSCs can inhibit immunogenic T cell activation by antigen-presenting cells, thus resulting in T cell apoptosis (25, 47). Collectively, these results indicate that HSCs significantly contribute to and participate in the intrahepatic immune microcircumstance by interacting with immune cells in a positive feedback manner (**Figure 6**).

## Conclusion

Immunosurveillance and immunoregulation of T cells are mainly dependent on TCR recognition (48). Our comprehensive scRNA-seq and TCR IR-seq databases revealed that TCR rearrangement does play a critical role in the regulation of the intrahepatic immune microcircumstance, which regulates HSC activation in the liver. Our observation of TCR IR reconstitution provides a novel setting for the fibrosis-associated immune microenvironment in response to liver damage. Additionally, it offers new insights into the immunosurveillance and immunoregulation of T cells.

## Data Availability Statement

The sequencing data has been deposited in the NGDC GSA database (https://bigd.big.ac.cn/gsa/; accession number: CRA003280).

## Ethics Statement

The animal study was reviewed and approved by Xiamen University Committee on Animal Care and Use.

## Author Contributions

KW and QYL conceived and designed the study. QL, MZ, CL, WZ, YL, PZ, and YC performed experiments. QYL and YH analyzed the scRNA-seq data. KW and PX analyzed the bulk IR-seq data. KW wrote this manuscript. All authors contributed to the article and approved the submitted version.

## Funding

This work was supported by the National Natural Science Foundation of China (81900569 and 82000592), the Fundamental Research Funds for the Central Universities (20720190080), the China National Postdoctoral Program for Innovative Talents (BX20190186), and the China Postdoctoral Science Foundation (2020M682093).

## Conflict of Interest

The authors declare that the research was conducted in the absence of any commercial or financial relationships that could be construed as a potential conflict of interest.
