# Supplementary material for: The T Cell Receptor Immune Repertoire Protects the Liver From Reconsitution
Source: Front Immunol. 2020 Dec 18;11:584979. doi: 10.3389/fimmu.2020.584979 (PMC7775400; doi:10.3389/fimmu.2020.584979)
Supplement: Supplementary file 1 [file DataSheet_1.docx]

**The T cell receptor immune repertoire protects the liver from fibrosis**

**Authors**

Qing Liang^1^, Yudi Hu^1^, Meina Zhang^1^, Chunjie Lin^2^, Wei Zhang^3^, Ying Li^4^, Ping Zhu^5^, Pengxin Xue^1^, Yujie Chen^1^, Qiyuan Li^1^, Kejia Wang^1*^

**Authors’ Affiliations**

^1^National Institute for Data Science in Health and Medicine, School of Medicine, Xiamen University, 361102 Xiamen, China. ^2^State Key Laboratory of Cellular Stress Biology, Innovation Center for Cell Signaling Network, School of Life Sciences, Xiamen University, 361102 Xiamen, China. ^3^Department of Pathology, The 971 Hospital of People’s Liberation Army Navy, 266071 Qingdao, China. ^4^Department of Pathology, Qingdao Municipal Hospital, 266011 Qingdao, China. ^5^Department of Gynaecology and Obstetrics, The 971 Hospital of People’s Liberation Army Navy, 266071 Qingdao, China.

Qing Liang and Yudi Hu contributed equally to this paper.

^*^**Correspondence to:**

Kejia Wang

Address: School of Medicine, Xiamen University, South Xiang’an Road, 361102 Xiamen, China.

E-mail: wangkejia@xmu.edu.cn

Tel/Fax.: +86-0592-2186786

**Supplementary Methods**

**Processing and quality control of scRNA-seq data**

The 10x Genomics pipeline CellRanger v.3.0.1 was used to process the raw sequence data with expect cells as 10,000. Reads were aligned to the mouse reference genome (mm10), filtered, and counted using ‘cellranger count’ to generate the gene-barcode matrix. The single-cell V(D)J sequences and clonotypes were processed using ‘cellranger vdj’ with standard settings. Expression matrices were processed using Seurat (v.3.0.0) R package. Briefly, the expression matrix was loaded using the ‘Read10X’ function. Quality control (QC) measures, including total unique molecular identifier (UMI) counts per cell, the number of genes detected per cell, and the percentage of mitochondrial transcripts per cell were calculated. Cells of each library were filtered to obtain distinct batch effects (Ctrl: nFeature > 500 and < 2500, nCount < 10,000, percentage of Mito genes < 6%; 4 W: nFeature < 2,200, nCount < 7,000, percentage of Mito genes < 6%; 8 W: nFeature < 2,000, nCount < 4,000, percentage of Mito genes < 10%). Cells within the QC addition were considered as good quality cells. Subsequently, the expression matrices of each library were merged for down-stream analysis.

**Dimensionality reduction**

The default log-normalization was performed using the ‘NormalizeData’ function. Cantering and scaling of the normalized values were done using the ‘ScaleData’ function with parameter ‘vars.to.regress’ as total UMI counts per cell. The number of genes detected per cell was used to regress out the confounders. A total of 500 most variable genes were identified using function ‘FindVariableGenes’ in the Seurat package. The identified highly variable genes were used as input to produce the Principle Component Analysis (PCA) projection using the ‘RunPCA’ function with standard settings. Next, the *t*SNE was performed using the ‘RunTSNE’ with the first 10 components from the PCA with default settings.

**Identification of immune cell types**

To identify cell types, we performed Shared Nearest Neighbor clustering (SNN) in the Seurat package and SingleR. The R package was based on an unbiased cell type recognition algorithm. Functions ‘FindNeighbor’ and ‘FindClusters’ were used to perform SNN clustering with a resolution of 0.5 in ‘FindClusters’. Raw count matrix was the inputted function ‘singler’ in the SingleR package with ‘immgen’ as reference. Subsequently, the marker genes in each cluster of the results from SNN and SingleR were identified. We integrated the results from two algorithms and identified 7 main cell types based on the marker genes of each cluster. T-cells were extracted as a single Seurat object, and the single-cell V(D)J sequences and clonotypes were integrated into it as metadata. The same SingleR pipeline was performed again to recognize the subtypes of T-cells with a higher resolution.

**Reconstructing single-cell trajectories**

The Monocle (v.2.10.1) package was used to reconstruct single-cell trajectories to discover developmental transitions among cells or time points. We used the T cells counts matrix as the input of monocle pipeline. Genes that passed the filter function ‘detectGenes’ with ‘min_expr’ = 0.1 and were expressed in more than 10 cells were selected. Next, the function ‘differentialGeneTest’ was used to identify the differential genes. The pseudo-time, which indicated the developmental process of each cell was calculated using ‘orderCells’ with the differential gene. ‘DDRTree’ was applied to reduce dimensions and visualize the pseudo-time process and bifurcations.

**TCRβ IR construction and data processing**

For the construction of the TCRβ IR, two-round nested amplicon arm-PCR was performed using specific primers according to the methods described previously (Liang et al., 2018). The PCR products were verified using agarose gel electrophoresis. The V, D and J segments of the TCRβ consensus sequences were identified using BLAST Plus in the international ImMunoGeneTics (IMGT) information system (IMGT: http://www.imgt.org/) by a standard algorithm.

**Flow cytometry and fluorescence-activated cell sorting (FACS)**

The isolated immunocytes were incubated for 20 minutes at 4°C in the dark with fluorescently conjugated antibodies directed against mouse CD45 (FITC, I3/2.3, Cat#: 147709), CD3e (APC, 145-2C11, Cat#: 100312), CD4 (FITC, clone: GK1.5, Cat#: 100405), CD8a (PE, clone: 53-6.7, Cat#: 100707), CD11b (FITC, clone: M1/70, Cat#: 101205), F4/80 (PE, clone: BM8, Cat#: 123109), NK1.1 (FITC, clone: PK136, Cat#: 108705), TCRβ (FITC, clone: H57-597, Cat#: 109205) and TCRγδ (APC, clone: GL3, Cat#: 118115) from Biolegend (San Diego, CA). Immune cell subsets were obtained from intrahepatic immunocytes by FACS. Regarding cytokine detection, the selected cells were incubated with antibodies against mouse IL-17 (FITC, clone: TC11-18H10.1, Cat#: 506907) and TNF-α (APC, clone: MP6-XT22, Cat#: 506307) (Biolegend, San Diego, CA) for 20 minutes at 4°C in the dark after incubating with permeabilization buffer. Next, the cells were stained and assessed using a BD FACSAria ІІ (BD Biosciences, Mountain View, CA, USA), then analyzed with FlowJo Version 10 software (TreeStar, Ashland, Oregon, United States). All data were acquired on a Helios mass cytometer.

**Mass cytometry**

Antibodies conjugation and cell labeling were conducted as described previously (Jia et al., 2020). Purified antibodies were purchased from Biolegend (San Diego, CA, USA) and labeled using the MaxPar DN3 labeling kits (Fluidigm Sciences, San Francisco, CA, USA) according to the manufacture’s protocol. The isolated immune cells (from WT, 4-week CCl_4_-treated liver, 4 mice per sample) were stained for viability test with 0.5 μM cisplatin (diluted in prewarmed serum-free medium) and then fixed with 1.6% paraformaldehyde for 10 minutes at room temperature. After washing with cell staining buffer (PBS with 0.5% BSA and 0.02% sodium azide), cells were incubated with Fc Receptor Blocking Solution for 10 minutes at room temperature. Subsequently, the cells were stained with antibodies against surface markers and intracellular markers overnight at 4°C. Afterwards, the cells were washed with cell staining buffer and DNA stained using 125 μM Cell-ID Intercalator Ir (Fluidigm Sciences) at room temperature for 1 hour. All data were acquired on a Helios mass cytometer.

**Processing of mass cytometry data**

The cytofkit (v.1.12.0) package in R was used to perform mass cytometry analysis. The raw fcs files were used as inputs of the function ‘cytof_exprs’ with ‘cytofAsinh’ as transformation method. A total of 50,000 cells were taken from each sample and merged into an integrated MFI matrix. The FlowSOM clustering algorithm was then performed to cluster different types of cells with FlowSOM_k = 20 using the function ‘cluster_FlowSOM_x’. Finally, *t*SNE was performed to reduce the dimensions and visualize the data.

**Quantitative real-time polymerase chain reaction (qPCR) assay**

Total RNA was isolated from liver tissues using TRIZOL reagent (Vazyme, Nanjing, Jiangsu, China), following the manufacturer’s instructions. The quantity and quality of the RNA were determined using a spectrophotometer (Eppendorf BioPhotometer Plus, Eppendorf, Hamburg, Germany). About 100 ng of RNA was reverse transcribed to cDNA using a Transciptor First Strand cDNA Synthesis Kit (Vazyme). Real-time qPCR was conducted using SYBR Green Reagent (Vazyme) on a T100^TM^ Thermal Cycler (Bio-Rad Inc., CA, USA). The relative gene expression was normalized to GAPDH and calculated using 2^−ΔΔCt^ method.

**Human liver specimens’ collection**

Paraffin-embedded human liver samples were obtained from the 971 Hospital of People’s Liberation Army Navy and Qingdao Municipal Hospital. The samples consisted of livers from patients who had autopsies or hepatectomy with hepatic pathological examination over a period of 5 years. Normal liver samples were collected from autopsy and exhibited no substantial pathological abnormalities. Cirrhotic patients with abnormal immune conditions, viral hepatitis, ongoing or advanced cancer, recent chemo- or radiotherapy, were excluded from the study. Ten normal liver samples and ten cirrhotic liver samples of both sexes were included for the analysis (The clinical characteristics shown in Table 1). This protocol (XM20180444) has been approved by the Hospital Medical Ethics Association of Xiamen University.

**Table 1. The clinical characteristics of patients.**

| **Group** | **Controls** | **Cirrhotic patients** |  |
| --- | --- | --- | --- |
| Age | 61.1±13.8 | 55.5±4.8 |  |
| Sex |  |  |  |
| Male | 8 | 7 |  |
| Female | 2 | 3 |  |
| Stage | NA |  |  |
| Child-Pugh B | NA | 3 |  |
| Child-Pugh C | NA | 7 |  |
| Albumin (g/L) | NA | 36.1±5.6 |  |
| Bilirubin (μmol/L) | NA | 19.2±9.1 |  |
| ALT (U/L) | NA | 160.2±178.2 |  |
| AST (U/L) | NA | 163.5±172.9 |  |

NA, not Applicable; ALT, alanine aminotransferase; AST, aspartate aminotransferase

**Histological and immunostaining assay**

The liver tissue samples embedded in paraffin were cut into 5-μm sections and dried overnight for haematoxylin and eosin (HE) staining. The staining was performed using Sirius red and Masson trichrome stains (Solarbio Life Sciences, Beijing, China). Immunofluorescence staining was performed as described previously (Liang et al., 2018). The following primary antibodies were used: CD3 (Abcam, clone: SP162, Cat#: ab135372), SMA (Abcam, clone: E184, Cat#: ab32575), and Ki67 (Abcam, clone: SP6, Cat#: ab16667). The liver fibrosis area fraction and immunocyte subsets were assessed in 5 cross-sections using Image-Pro Plus 6.0 software (Media Cybernetics, Rockville, MD, USA).

**Isolation of HSCs and co-culture**

Primary HSCs were isolated as described previously with a few modifications (Oben et al., 2003). Briefly, the liver tissue was first perfused in situ with D-Hanks (Solarbio Life Sciences, Beijing, China) containing 25 U/ml heparin, then with D-Hanks containing pronase (0.05%), Ⅳ collagenase (1%) and DNase Ⅰ (1%) until the liver lost its firm texture. The liver was excised, placed in D-Hanks containing pronase, Ⅳ collagenase, and DNase Ⅰ at 37℃ for 10 minutes. Next, the homogenate was filtered through a 75-μm gauge stainless steel mesh. The suspension was centrifuged (40 × g) for 3 minutes at 4℃, transferred to a new tube, then centrifuged (580 × g) for 8 minutes at 4℃. After the supernatant was removed, the pelleted cells were resuspended in 50% and 25% Percoll in D-Hanks containing 100 U/ml heparin, then centrifuged (900 × g) for 30 minutes at 4℃. The interface was gently aspirated, mixed with Dulbecco's Modified Eagle Medium (HyClone, Logan, UT, USA) and centrifuged at 400g for 10 minutes at 4℃. For co-culture experiments, the isolated HSCs were cultured in a 12-well plate (1 × 10^5^ cells/well) with RPMI 1640 medium (Sangon Biotech, Shanghai, China) containing penicillin and streptomycin, and 20% fetal bovine serum (HyClone, Logan, UT, USA) at 37°C for 24 hours. Co-cultures were obtained by adding isolated intrahepatic T-cells (CD3e^+^ and TCRβ^+^, 1 × 10^4^ cells) to the HSCs culture plates. RPMI 1640 medium with 5 ng/ml IL2, 20 ng/ml IL12, and 20 ng/ml IL18 (Carrier free, R&D Systems) was used to achieve T-cell activation, and co-cultured cells were maintained for 4 days. The cell viability was measured using Cell Counting Kit-8 (CCK-8, Dojindo, Kumamoto, Japan). Unless otherwise mentioned, all the chemicals/reagents used in this section were purchased from Solarbio Life Sciences.

**Supplementary Figures**


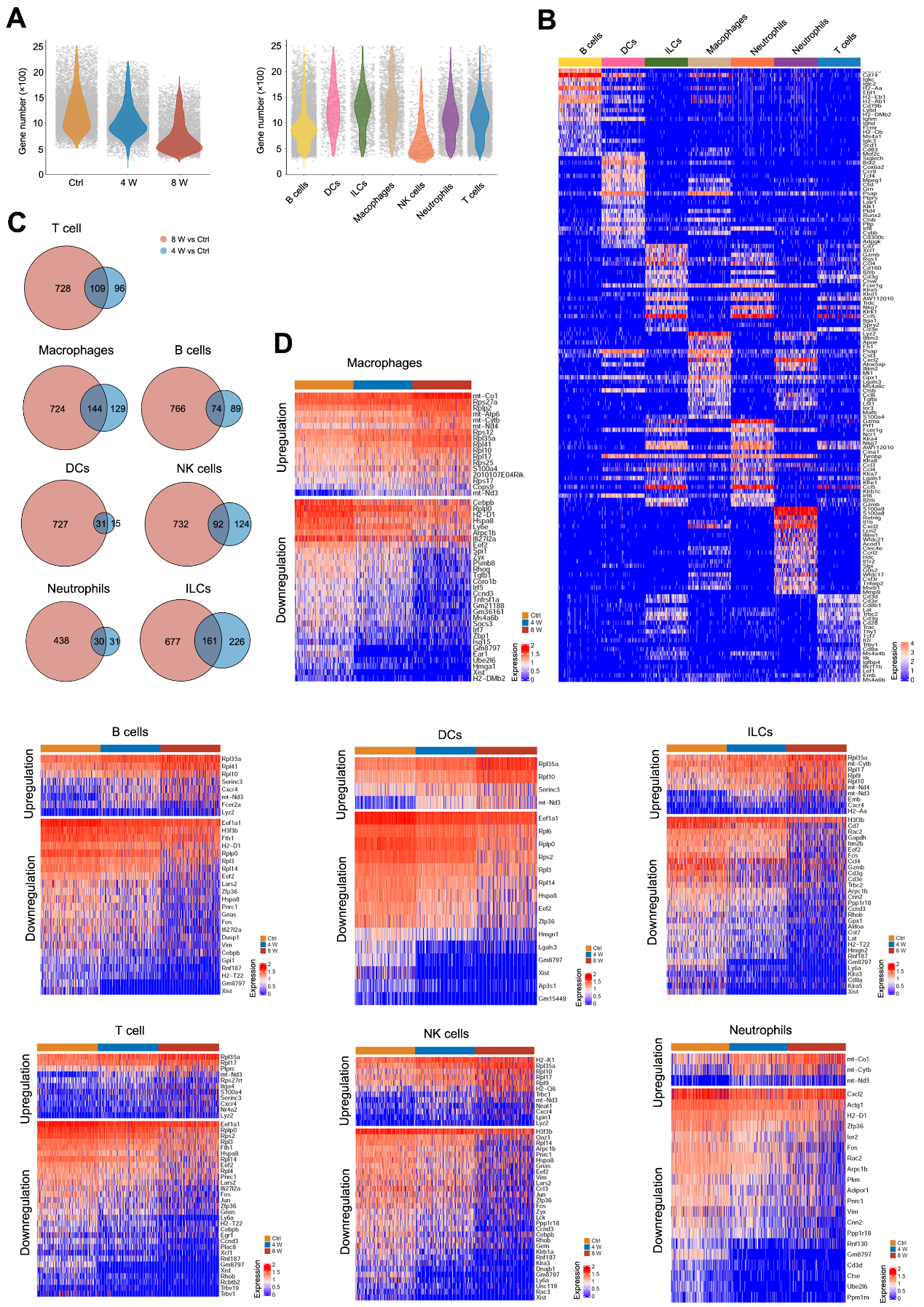


**Figure S1.** **Identification of intrahepatic immune microcircumstance for Single-Cell Atlas in liver fibrosis. (A)** Unique gene counts of 28,608 single-cell transcriptomes, separated by group (left) or cell type (right). **(B)** Heat map showing the expression of marker genes of each cell type identified by Seurat (complete data available in Table S1). **(C)** Venn diagrams showing the number of DEGenes in each cell type in the indicated groups. **(D)** A heatmap showing Z-scored mean expression of DEGenes of each cell type (complete DEGenes available in Table S2).


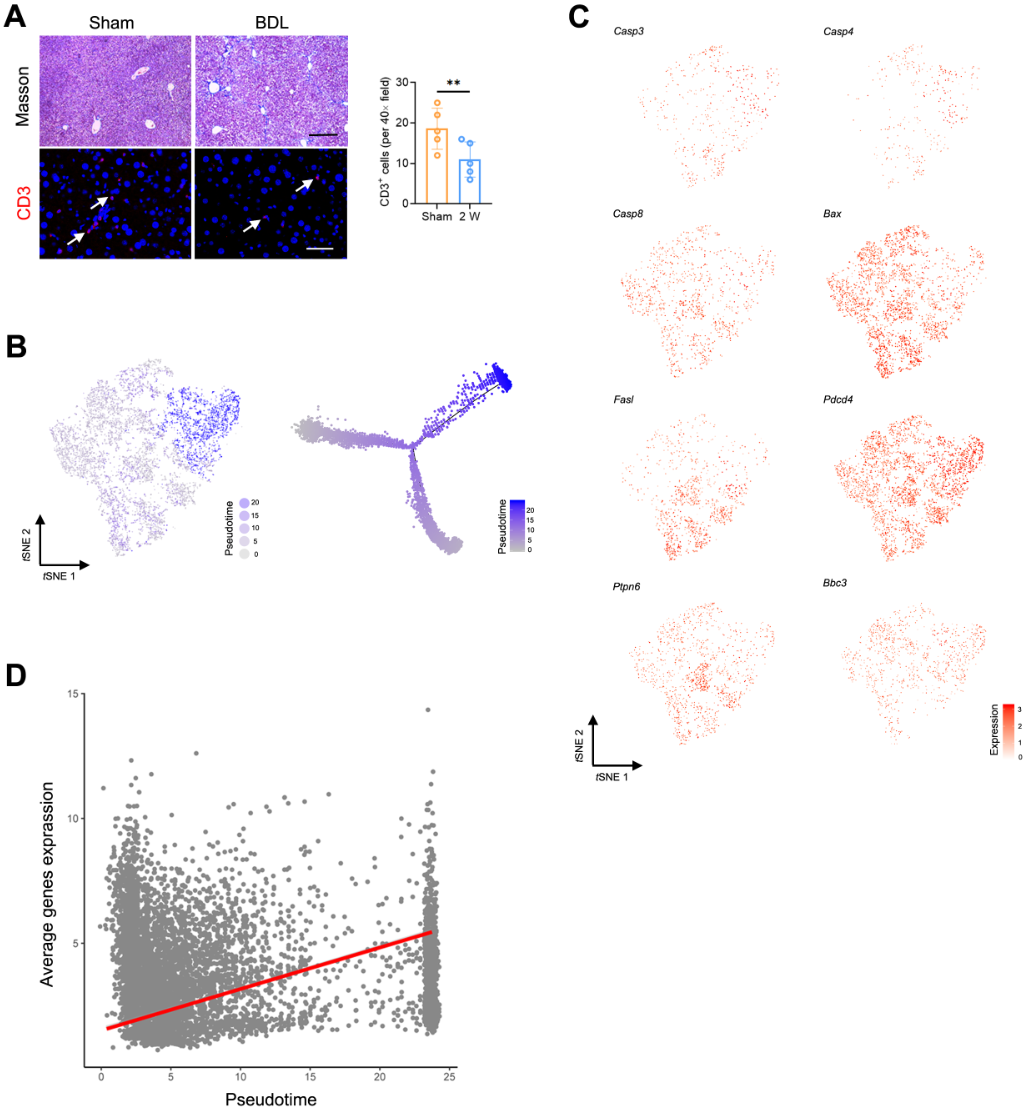


**Figure S2.** **T cell transition during liver fibrosis.** **(A)** Representative Masson trichrome and immunofluorescence staining of liver tissues from sham and BDL mice 2 week after laparotomy. Scale bar, 100 μm. **(B)** Trajectory analysis from T cell scRNA-Seq data in two-dimensional state-space inferred by Monocle 2. **(C)** *t*SNE plot coloured with single-cells with a high expression of genes shown in red, and those with a low frequency shown in grey. **(D)** Traceplot of T cell apoptosis signature (defined as mean expression across genes in signature in **(C)** for all T cells. X axis is projected along pseudotime, and the red line indicates linear regression of the signature expression (*r* = 0.0024, *P* < 0.0001).


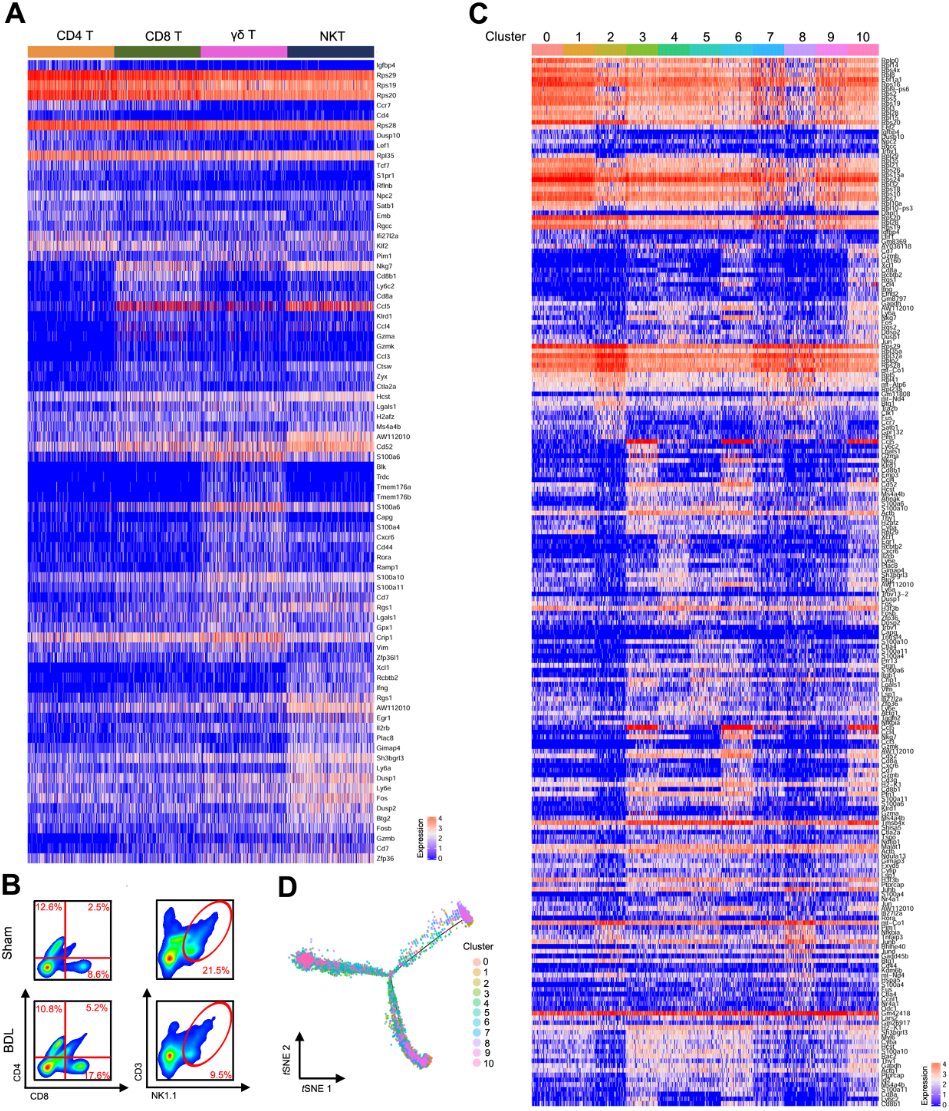


**Figure S3.** **Identification of intrahepatic T cells for Single-Cell Atlas. (A)** A heatmap showing the expression of marker genes of CD4 T, CD8 T, γδ T and NKT cells (complete data available in Table S4). **(B)** Flow cytometry for CD4 T cells (CD4^+^), CD8 T cells (CD8^+^) and NKT cells (NK1.1^+^, CD3e^+^) in sham and BDL groups. 10,000 cells for each sample. **(C)** Heat map showing the expression of marker genes for 10 clusters (complete data available in Table S6). **(D)** Trajectory analysis of T cell clusters in two-dimensional state-space inferred by Monocle 2.


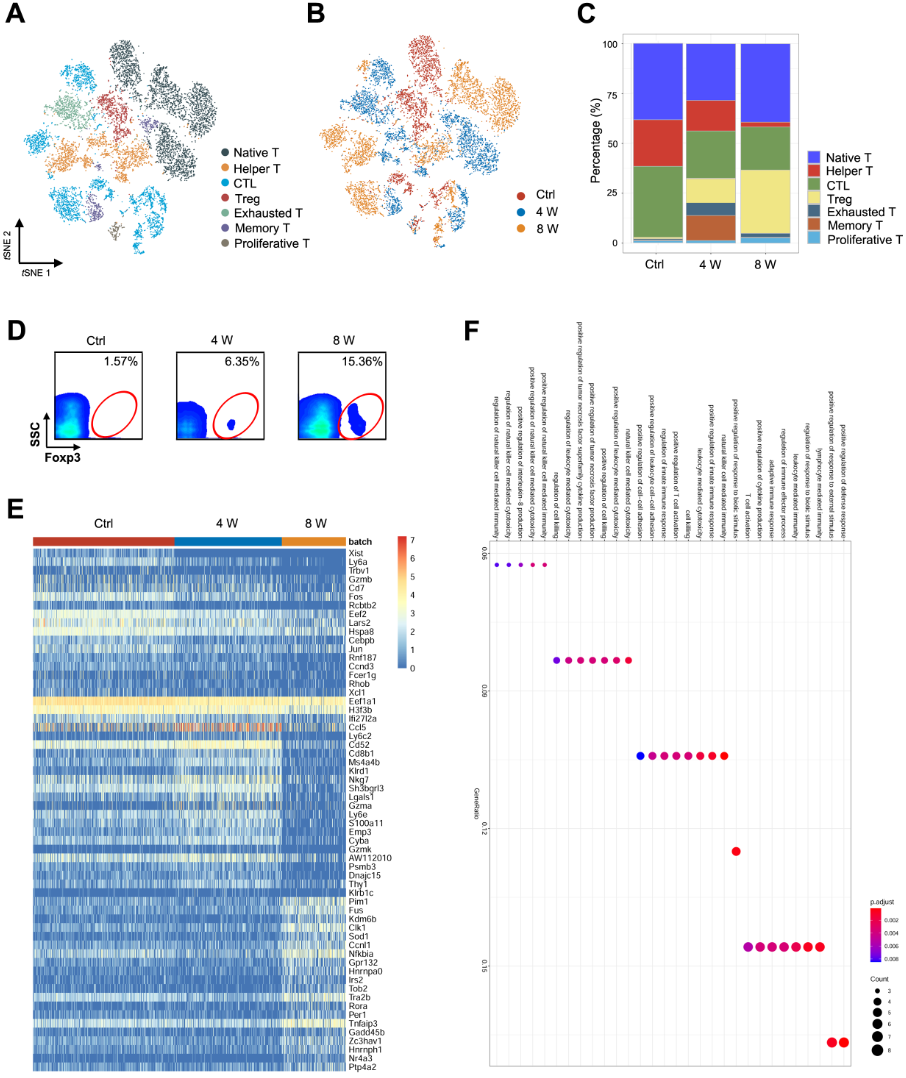


**Figure S4. Change of Treg during liver fibrosis.** **(A)** *t*SNE plot coloured by different T cell subsets (native T cells, helper T cells, cytotoxic T cells (CTL), regulatory T cells (Treg), exhausted T cells, memory T cells, proliferative T cells). **(B)** *t*SNE plot of αβ T cell coloured by groups. **(C)** The percentage of different T cell subsets. **(D)** Flow cytometry for Treg cells (Foxp3^+^) in untreated, 8-week CCl_4_-treated livers. **(E)** Heatmap showing DEGenes of Treg. (Complete DEGenes available in Table S5). **(F)** Pathway enrichment for the DEGenes (4 W).

**
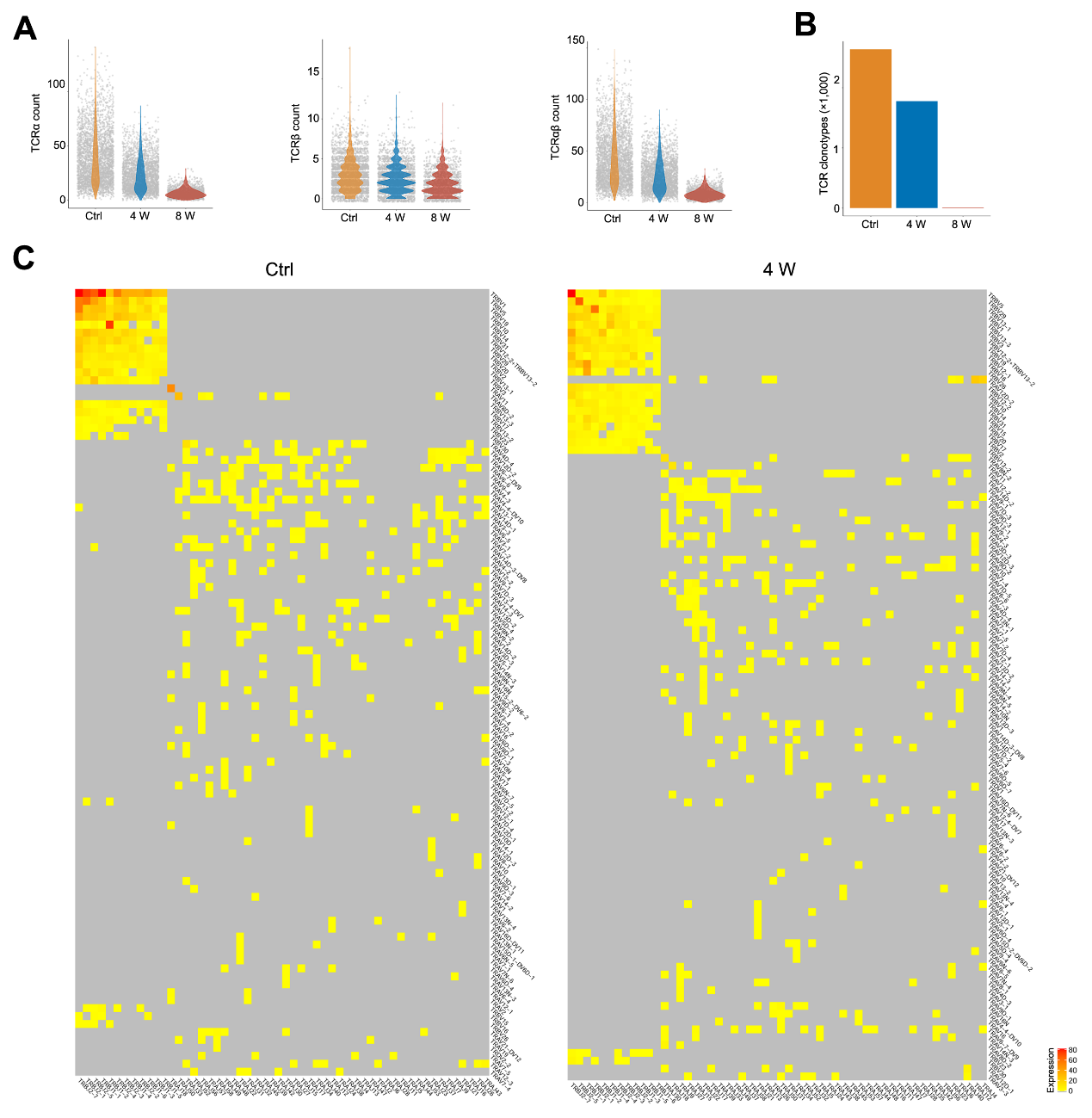
**

**Figure S5.** **Paired single-cell TCR identification. (A)** Counts of TCRα (left), TCRβ (middle) and TCRαβ (right) identified from paired single-cell sequencing. **(B)** A total of TCR clonotypes of scRNA-seq data in the indicated groups. **(C)** A heatmap showing Z-scored mean expression of Ctrl (left) and 4 W (right) VJ combination usage (complete data available in Table S9).

**
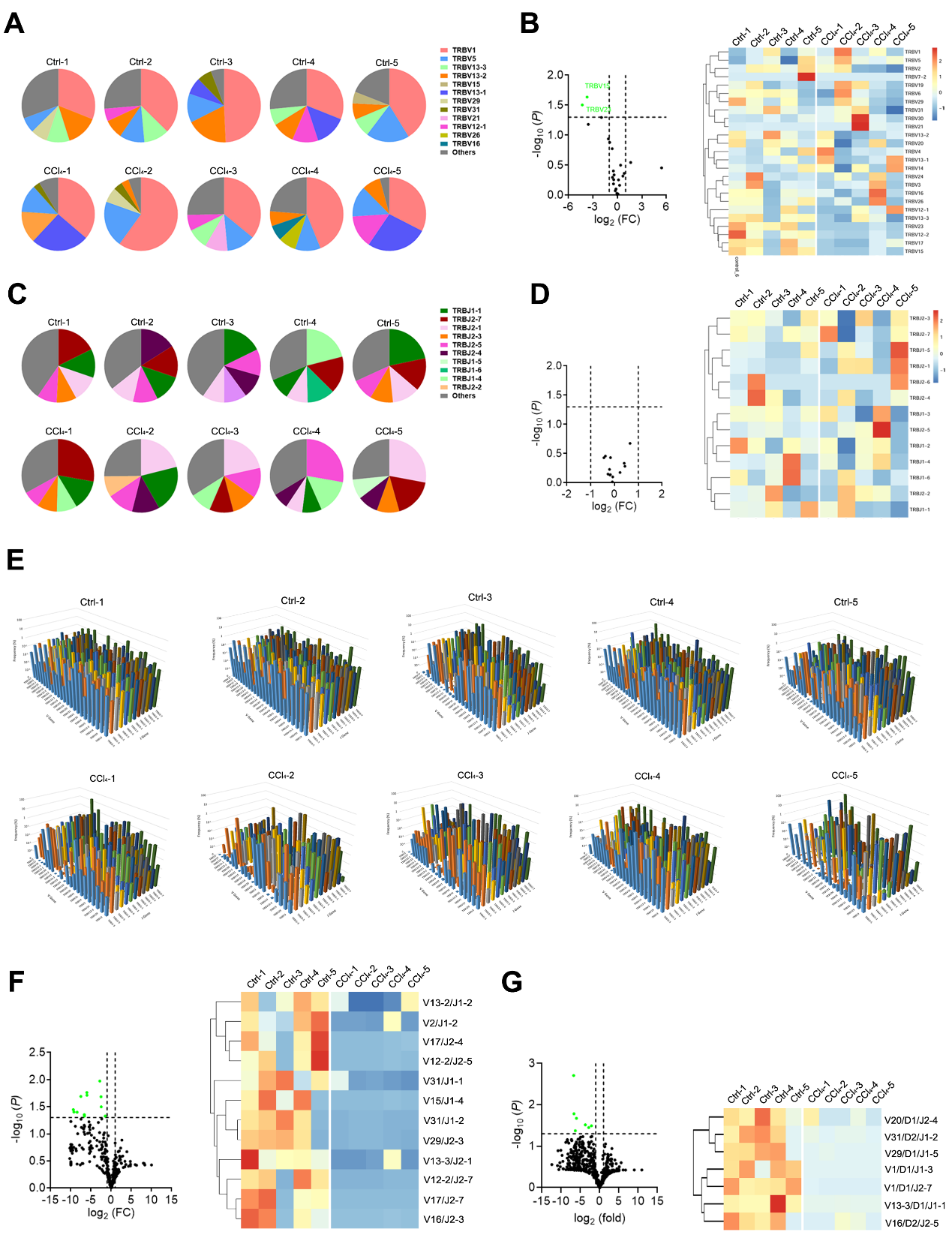
**

**Figure S6.** **The profile of TCRβ V, D and J segments.** TCRβ IR-seq of intrahepatic immune cells in the indicated groups. **(A)** Distribution of the top 5 TRBV segments in each sample. **(B)** Volcano plot showing TRBV segments of 8 W *vs.* Ctrl (left). A heatmap showing Z-scored mean expression of TRBV segments (right). **(C)** Distribution of the top 5 TRBJ segments in each sample. **(D)** A Volcano plot showing TRBJ segments of 8 W *vs.* Ctrl (left). A heatmap showing the Z-scored mean expression of TRBJ segments (right). **(E)** The frequency distribution of VJ combinations in each sample. **(F)** A Volcano plot showing VJ combination of 8 W *vs.* Ctrl (left). A heatmap showing Z-scored mean expression of differential VJ combination (right). **(G)** A Volcano plot showing VDJ combination of 8 W *vs.* Ctrl (left). A heatmap showing Z-scored mean expression of differential VDJ combination (right). Green dots indicate significant downregulated segments. Complete data provided in Table S10.

**
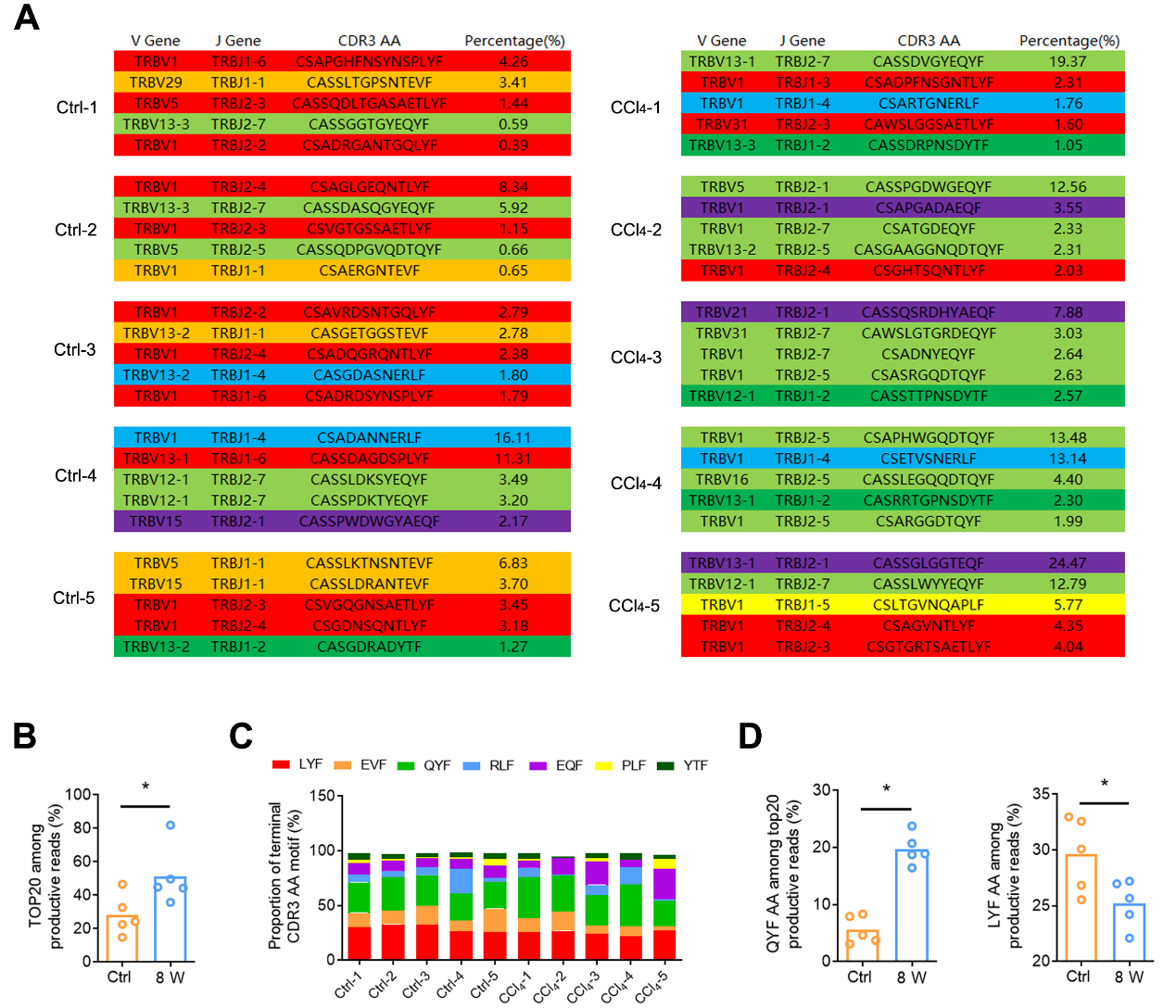
**

**Figure S7.** **Reconstitution of TCRβ CDR3 AA in fibrotic liver.** **(A)** Fraction of the top 5 TCRβ V-CDR3-J clonotypes in each sample (complete data in Table S12). **(B)** Proportion of the top 20 clonotypes for V-CDR3-J. **(C)** Fraction of the terminal primary CDR3 AA motif in each sample. Colours indicate different terminal CDR3 AA motifs (each colour represents a clonotype). **(D)** Proportion of the terminal CDR3 AA motifs of QYF and LYF (complete data in Table S13). Experiments were performed with 5 mice per group. *, *P*<0.05.

**
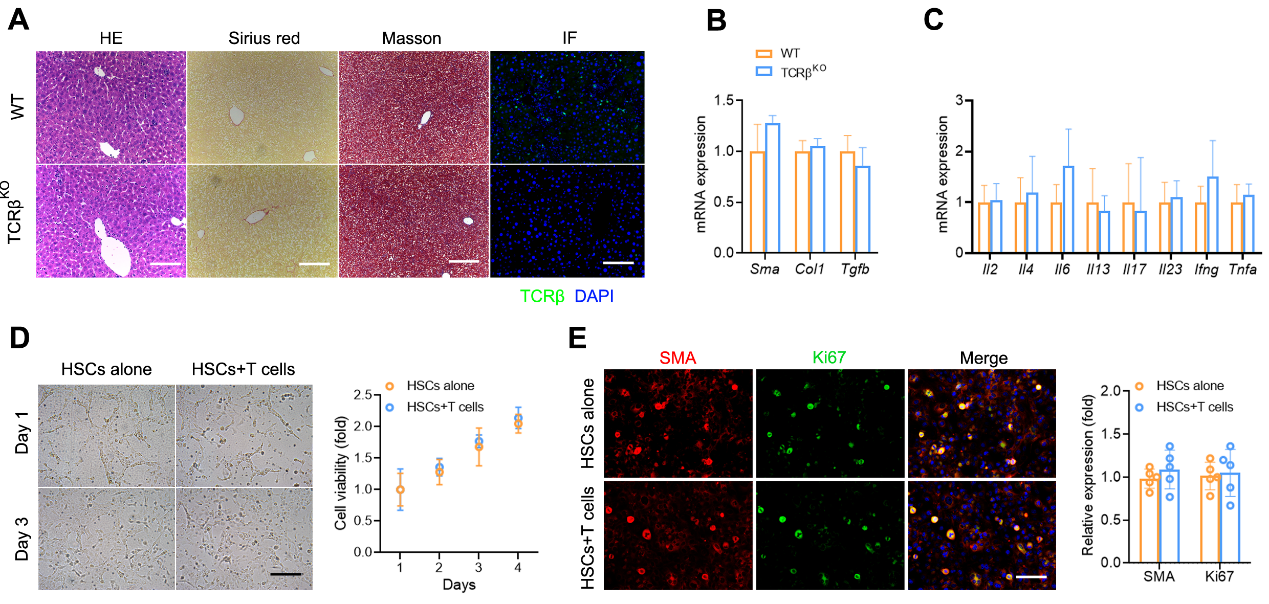
**

**Figure S8.** **The effect of TCRβ on HSCs *in vivo* and *in vitro*. (A)** Representative HE, Masson trichrome, Sirius red and immunofluorescence staining of liver tissues from WT and Tcrb^KO^ mice before CCl_4_ treatment. Scale bar, 100 μm. **(B)** qPCR analysis of mRNA level of fibrosis-related genes *Sma*, *Col1* and *Tgfb* in the liver after CCl_4_ treatment. **(C)** qPCR analysis of mRNA levels of inflammation-related genes *Il2*, *Il4*, *Il6*, *Il13*, *Il7*, *Il23*, *Ifng*, *Tnfa*. **(D)** Co-cultures of primary HSCs (isolated from 4-weeks CCl_4_-treated liver) with or without intrahepatic T cells (isolated from WT mice liver) for 4 days *in vitro*. Representative images showing day 1 and day 3 co-cultured cells (left). Scale bar, 50 μm. Cell viability was measured using the CCK-8 assay (right). **(E)** immunofluorescence staining for SMA (red) and Ki67 (green) performed to identify the proliferating HSCs. Representative images are shown. Scale bar, 50 μm. Quantification of expression of SMA and Ki67 in 10 cross-sections. Experiments were performed with 4-8 mice per group.

**
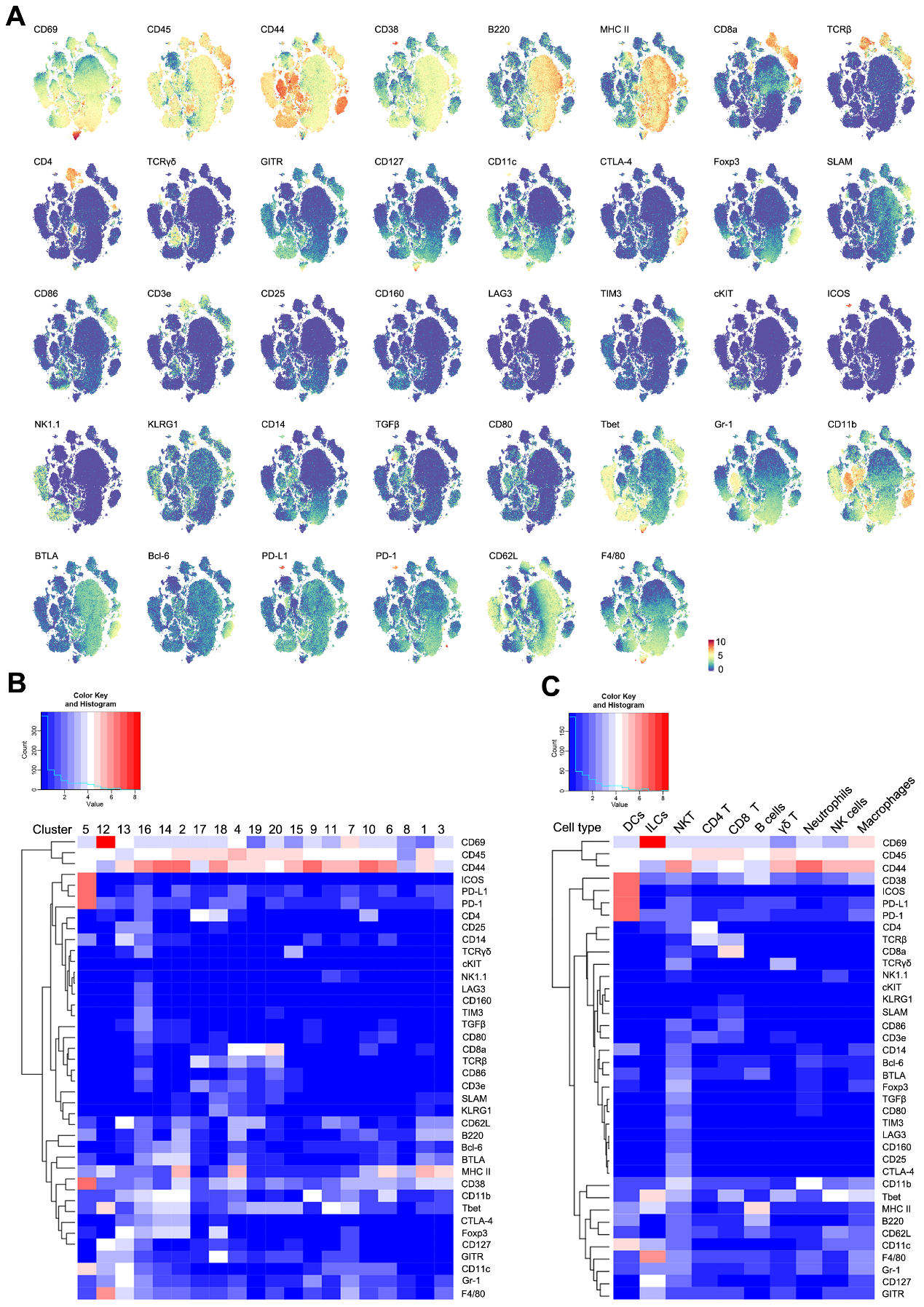
**

**Figure S9. Mass cytometric analysis of intrahepatic immune cells. (A)** *t*SNE clustering of 100,000 immune cells in liver tissues from WT (50,000, n = 4) and Tcrb^KO^ (50,000, n = 4) mice. Colours represent the expression values of the indicated markers. **(B)** A heatmap showing the expression of markers of 20 clusters. **(C)** A heatmap showing the expression of markers of each cell type. Complete data provided in Table S14.

**References**

Jia, X., Zhou, X., Zheng, H., Jiang, S., Weng, J., Huang, L., Du, Z., Xiao, C., Zhang, L., Chen, X.L., and Fu, G. (2020). A Carrier Strategy for Mass Cytometry Analysis of Small Numbers of Cells. *Methods Mol Biol* 2111**,** 21-33.

Liang, Q., Liu, Z., Zhu, C., Wang, B., Liu, X., Yang, Y., Lv, X., Mu, H., and Wang, K. (2018). Intrahepatic T cell receptor beta immune repertoire is essential for liver regeneration. *Hepatology*.

Oben, J.A., Yang, S., Lin, H., Ono, M., and Diehl, A.M. (2003). Acetylcholine promotes the proliferation and collagen gene expression of myofibroblastic hepatic stellate cells. *Biochem Biophys Res Commun* 300**,** 172-177.
